# Supplementary material for: Structural diversity in three-dimensional self-assembly of nanoplatelets by spherical confinement
Source: Nat Commun. 2022 Oct 12;13:6001. doi: 10.1038/s41467-022-33616-y (PMC9556815; doi:10.1038/s41467-022-33616-y)
Supplement: Supplementary file 1 — Supplementary Information [file 41467_2022_33616_MOESM1_ESM.pdf]

Structural diversity in three-dimensional self-assembly of nanoplatelets by spherical confinement

Da Wang[1,2,‖,*], Michiel Hermes[1,‖], Stan Najmr[3], Nikos Tasios[1], Albert Grau-Carbonell[1], Yang Liu[1,4,†], Sara Bals[2], Marjolein Dijkstra[1], Christopher B. Murray[3,5], and Alfons van Blaaderen[1,*]

[1]*Soft Condensed Matter, Debye Institute for Nanomaterials Science, Utrecht University, Princetonplein 5, 3584 CC, Utrecht, The Netherlands.*

[2]*Electron Microscopy for Materials Science (EMAT), University of Antwerp, Groenenborgerlaan 171, 2020 Antwerp, Belgium.*

[3]*Department of Chemistry, University of Pennsylvania, Philadelphia, PA 19104, United States.*

[4]*Department of Earth Sciences, Utrecht University, Budapestlaan 4, 3584 CD Utrecht, The Netherlands.*

[5]*Department of Materials Science and Engineering, University of Pennsylvania, Philadelphia, PA 19104, United States.*

[†]*Present address: Monash Centre for Electron Microscopy, Monash University, Clayton, VIC 3800, Australia.*

[‖]*These authors contributed equally to this work.*

[*]*Corresponding authors: dawangcolloid@gmail.com; a.vanblaaderen@uu.nl*

Supplementary Figures

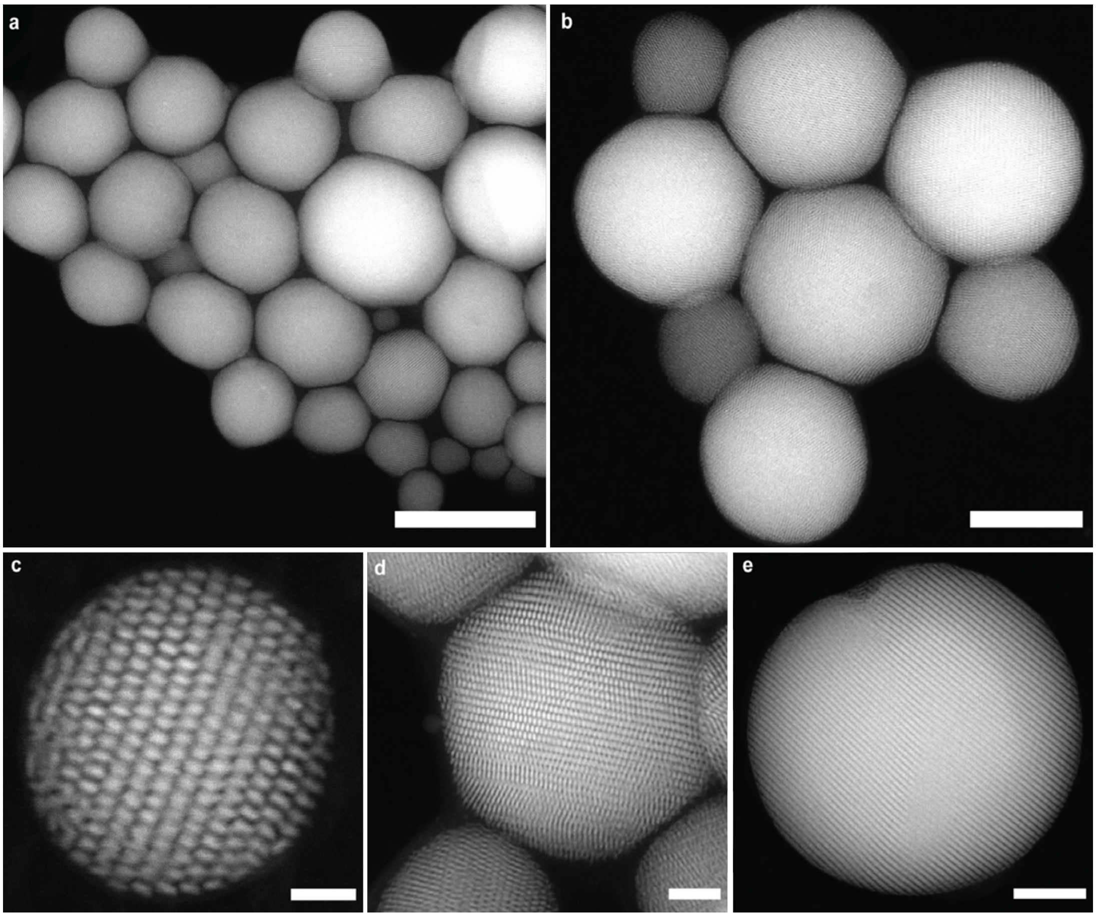

Supplementary Figure 1: Morphology of self-assembled supraparticles (SPs) composed of disk-shaped EuF_3 nanoplatelets (NPLs). a) Overview, b) a zoomed-in view of the self-assembled SPs. Three SPs with a diameter of c) 100 nm, d) 290 nm and e) 460 nm, respectively, showing that the NPLs stacked into straight columns. The outside NPLs oriented their edges towards the confining interface as can be seen in panels c-d. Scale bars, a) 500 nm, b) 200 nm and c) 20 nm, d) 50 nm, e) 100 nm. Note that a deviation from a spherical geometry in some SPs can be ascribed to capillary forces that are due to the water films in between the SPs in combination with plasticity of the NPL ensemble which was caused by the relatively high volume fraction of the ligands in the SP system.

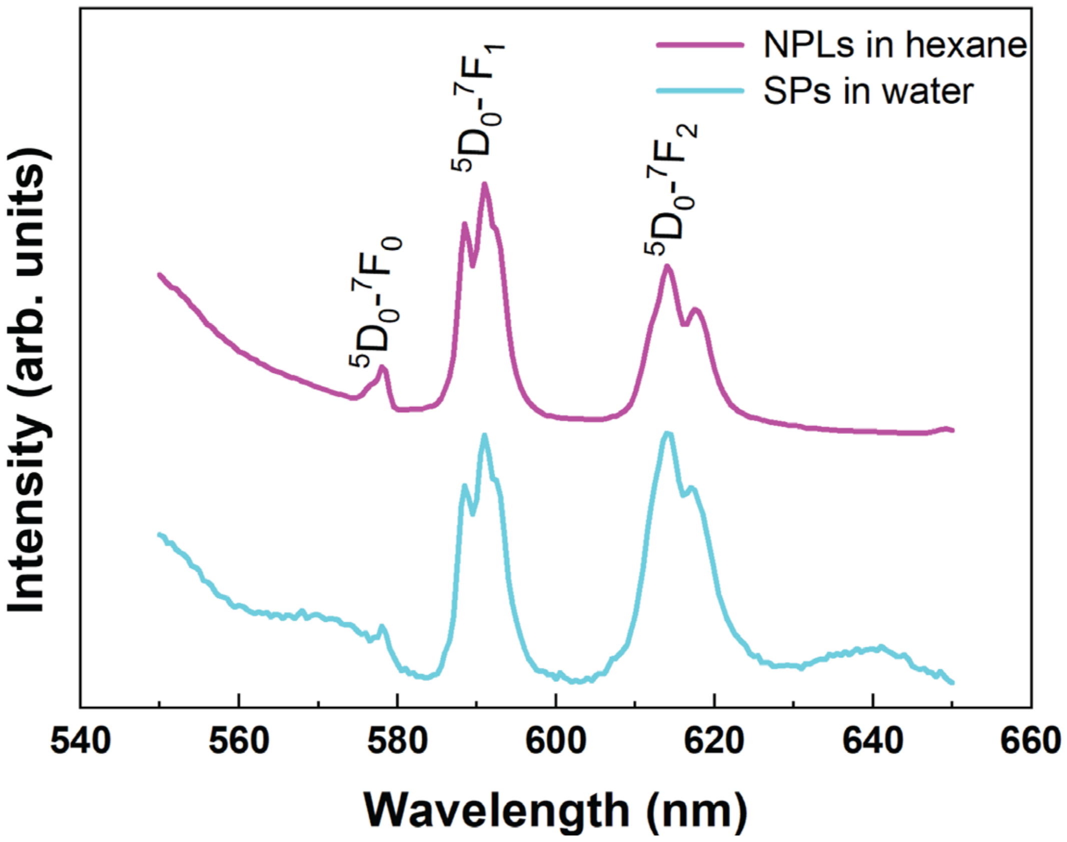

Supplementary Figure 2: Emission spectra of a dispersion of free EuF_3 NPLs in hexane and self-assembled SPs in water. Excitation wavelength was set at 395 nm. Excitation spectrum was recorded by monitoring the emission at 592 nm and 615 nm. See Supplementary Notes for details.

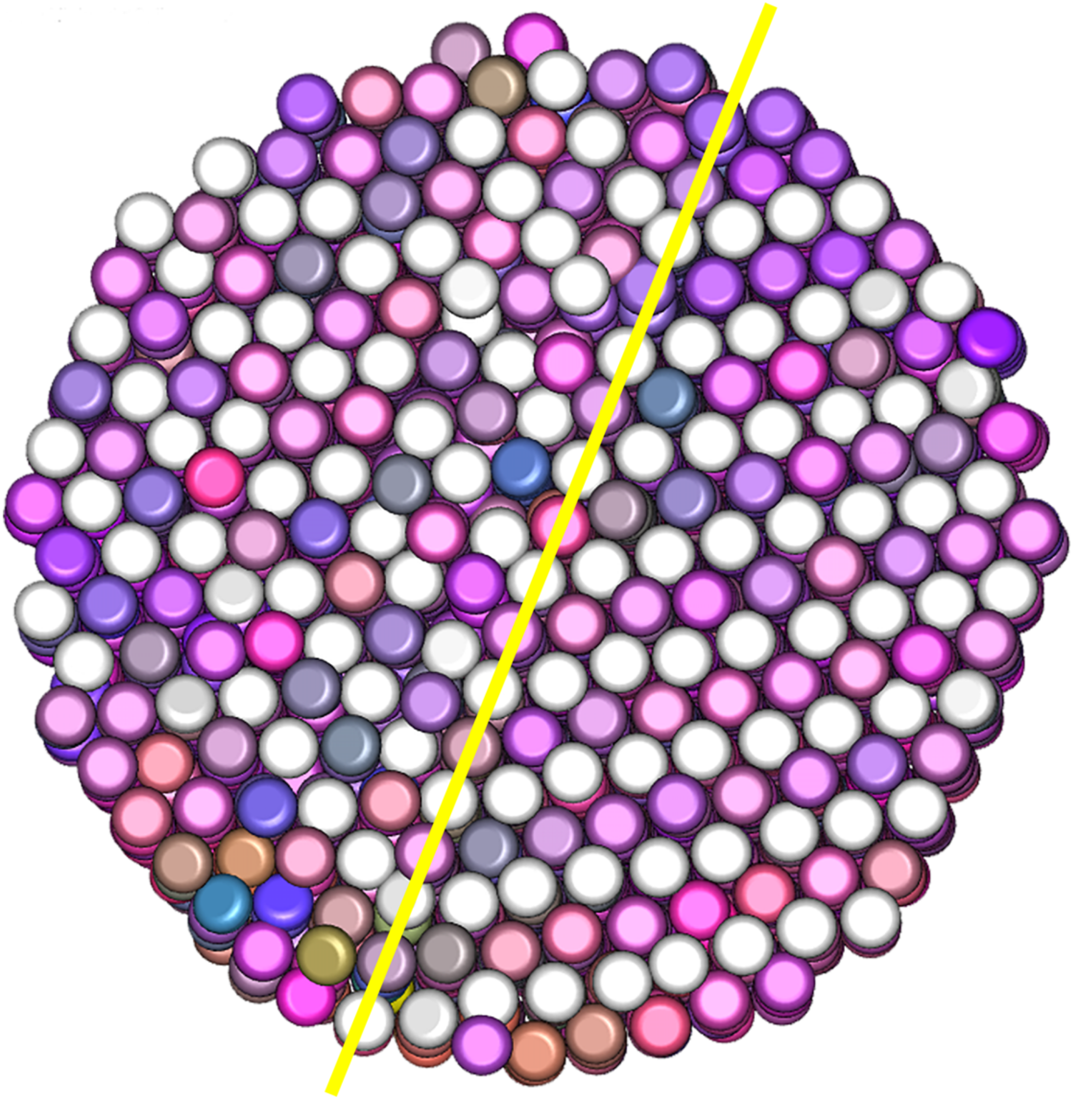

Supplementary Figure 3: Twinning defect of the disk-shaped NPLs in a self-assembled SP. Twinning boundary as denoted by a yellow solid line.

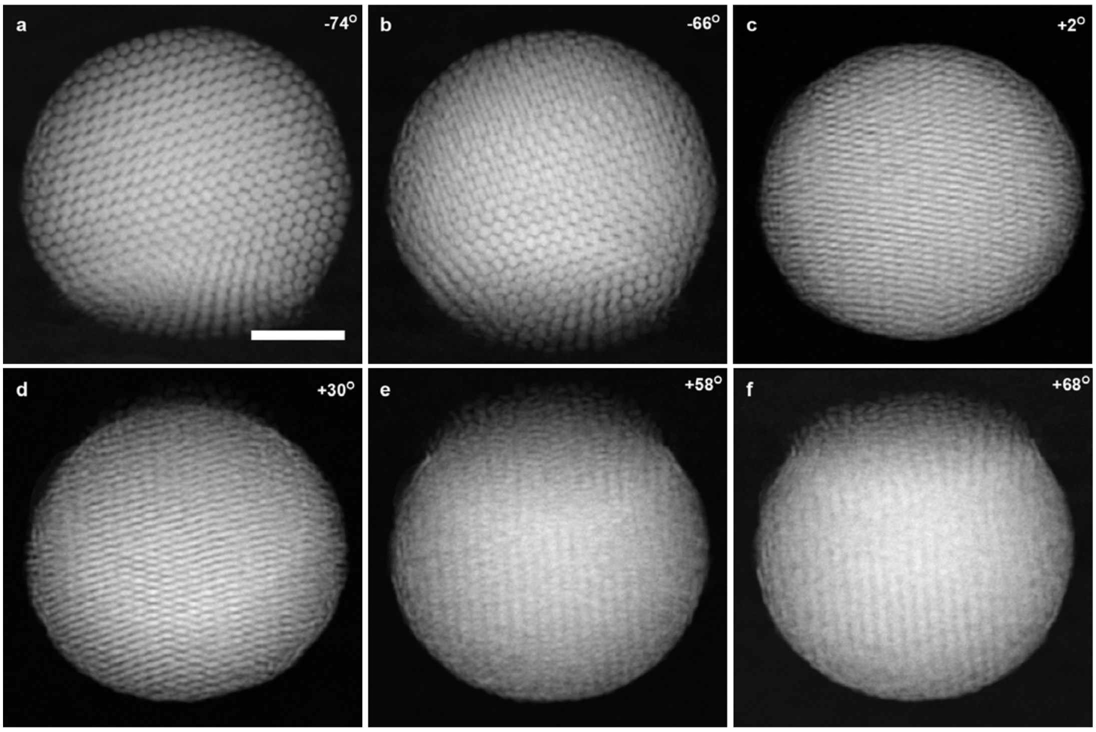

Supplementary Figure 4: 2D electron microscopy (EM) images of a single SP with a diameter of 183 nm obtained at different tilting angles. High-angle annular dark-field scanning transmission electron microscopy (HAADF-STEM) images of a SP with a diameter of 183 nm obtained at a series of tilting angles: a) $-74°$, b) $-66°$, c) 2°, d) 30°, e) 58° and f) 68°, showing either flat faces or edges of the disk-shaped EuF_3 NPLs oriented towards the spherical confining interface. Scale bar, 50 nm.

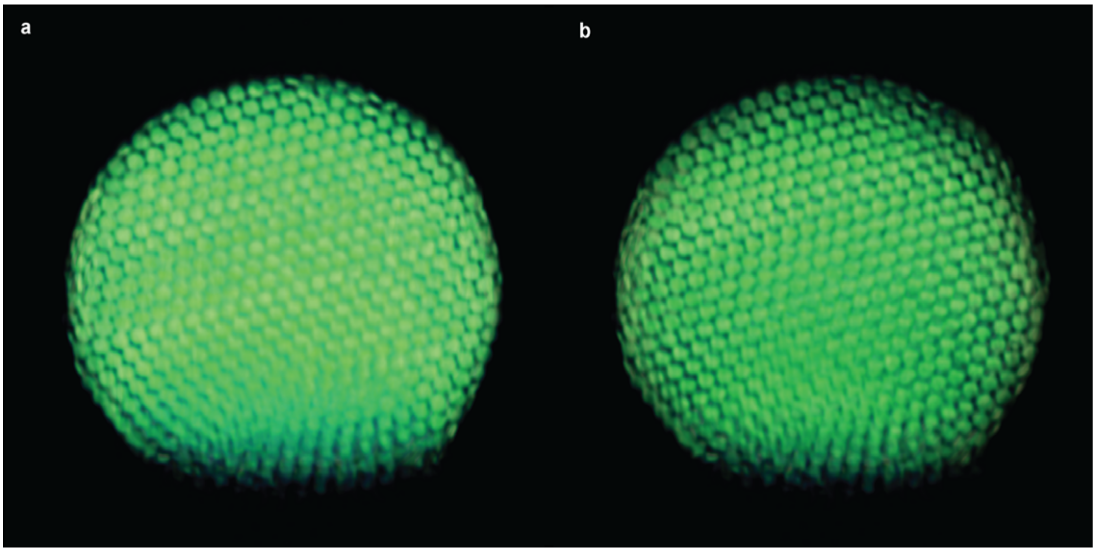

Supplementary Figure 5: 3D representation of a SP with a diameter of 183 nm consisting of disk-shaped EuF_3 NPLs. a) Overall and b) cut-through view of the SP with orientation of NPLs aligning their flat faces towards the confining interface, viewed along the column stacking direction. Note that the transparency was increased for visual clarity.

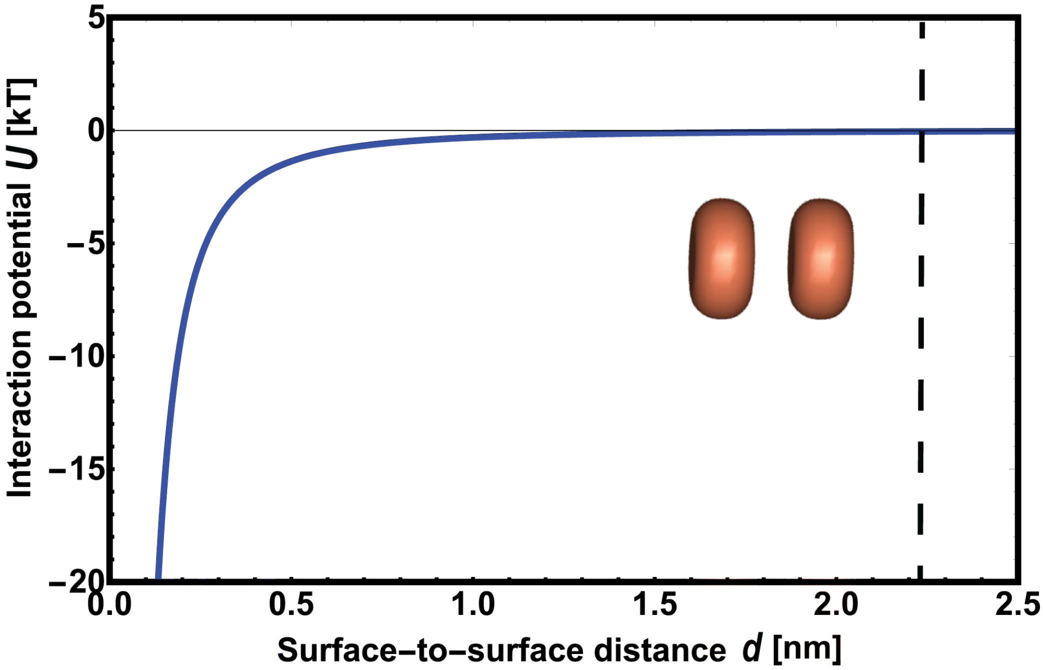

Supplementary Figure 6: van der Waals (vdW) interaction potentials between two EuF_3 NPLs. vdW potentials were calculated for two disk-shaped EuF_3 NPLs with a face-to-face orientation. The vertical dashed line denotes the closest interparticle distance between the two flat faces of two disk-shaped EuF_3 NPLs, demonstrating that the NPLs interact through a hard-particle potential.

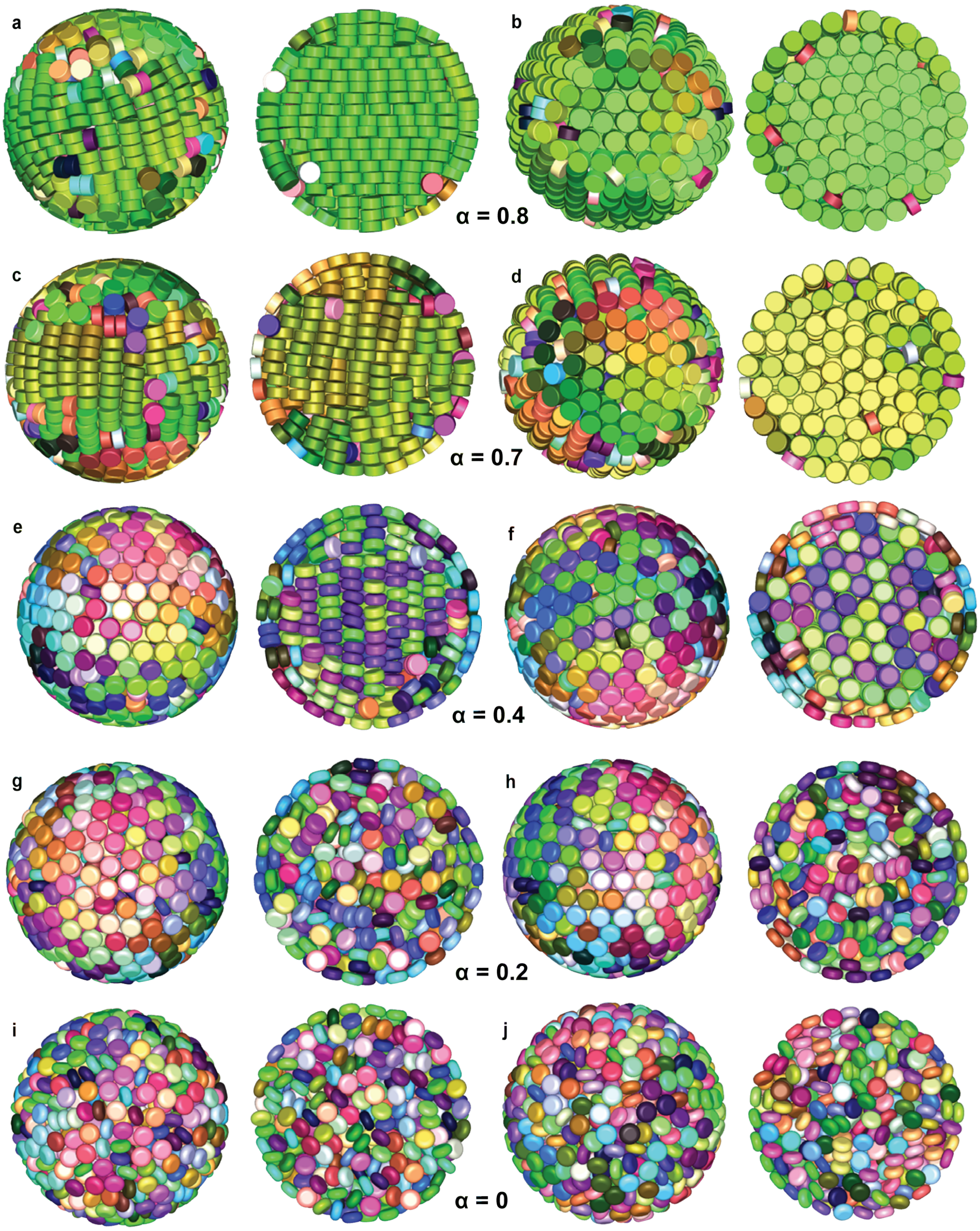

Supplementary Figure 7: Clusters obtained from computer simulations of hard disk-shaped particles with varying rounding parameters. Fully compressed configurations as obtained from computer simulations with 1,000 platelets in spherical confinement with different rounding parameter α. a-b) $\alpha = 0.8$, c-d) 0.7, e-f) 0.4, g-h) 0.2 and i-j) 0 (oblate hard spherocylinders), respectively. Note that we show the structure of each cluster from two different orientations. Different colours represent different particle orientations. The left and right column of each pair, shows a cut-through and surface view of each simulated cluster. For all NPLs, $h = 0.5L$. For an interactive 3D view, see Supplementary Data 3, 4, 7, 9 and 11, respectively.

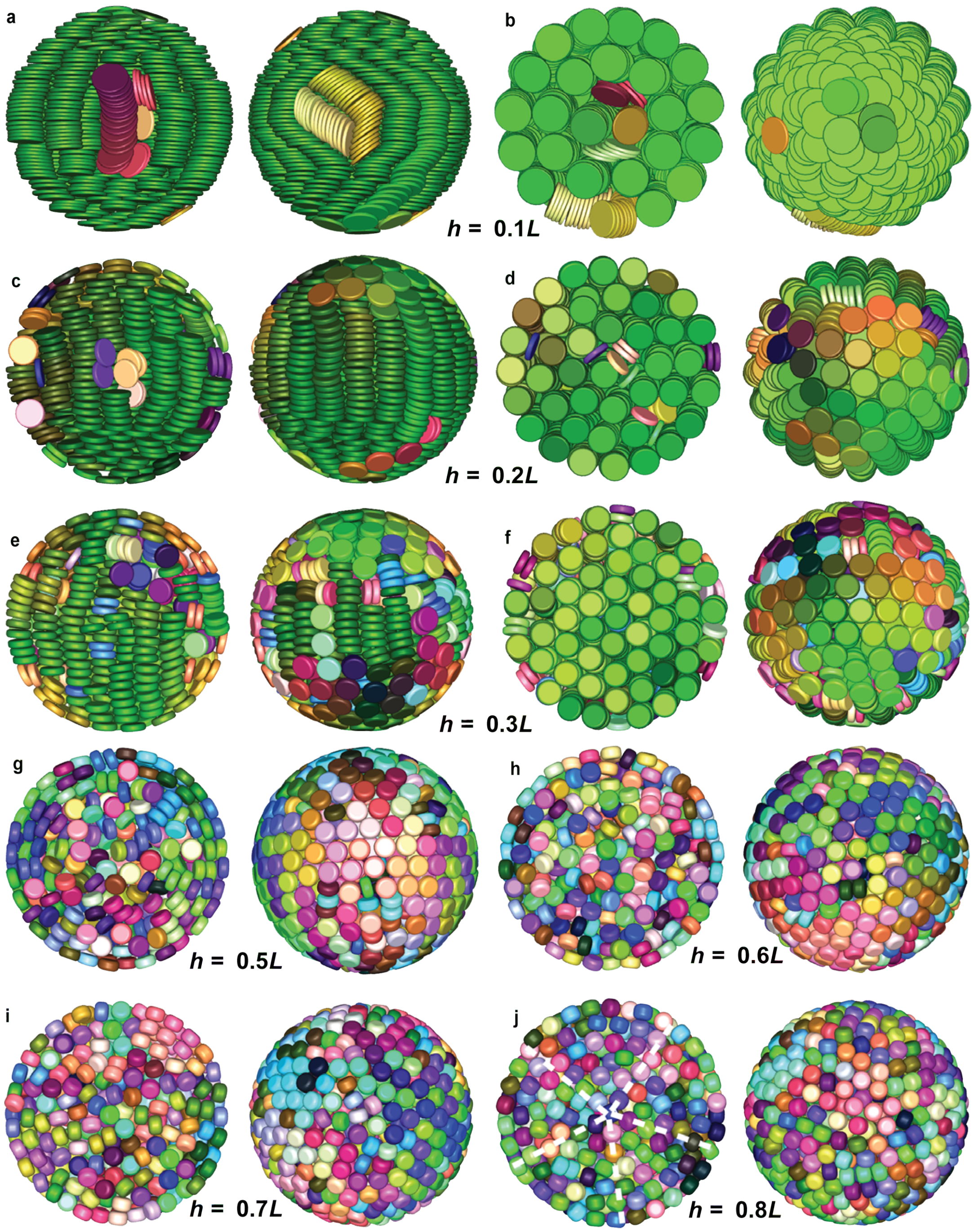

Supplementary Figure 8: Clusters obtained from computer simulations of hard disk-shaped particles with varying aspect ratios. Fully compressed configurations as obtained from computer simulations with 1,000 platelets in spherical confinement with different aspect ratio h/L. a-b) 0.1, c-d) 0.2, e-f) 0.3, g) 0.5, h) 0.6, i) 0.7 and j) 0.8, respectively. Different colours represent different particle orientations. The left and right column of each pair, shows a cut-through and a surface view of each simulated cluster. For all NPLs, $\alpha = 0.3$. A simulated Mackay icosahedral cluster shows a five-fold symmetry denoted by white dashed lines (aspect ratio $h/L = 0.8$; Supplementary Fig. 8j). For an interactive 3D view, see Supplementary Data 12, 13, 14, 8, 16, 17 and 18, respectively. The five-fold symmetry of the icosahedral cluster can be appreciated from the FFT pattern in Supplementary Data 18.

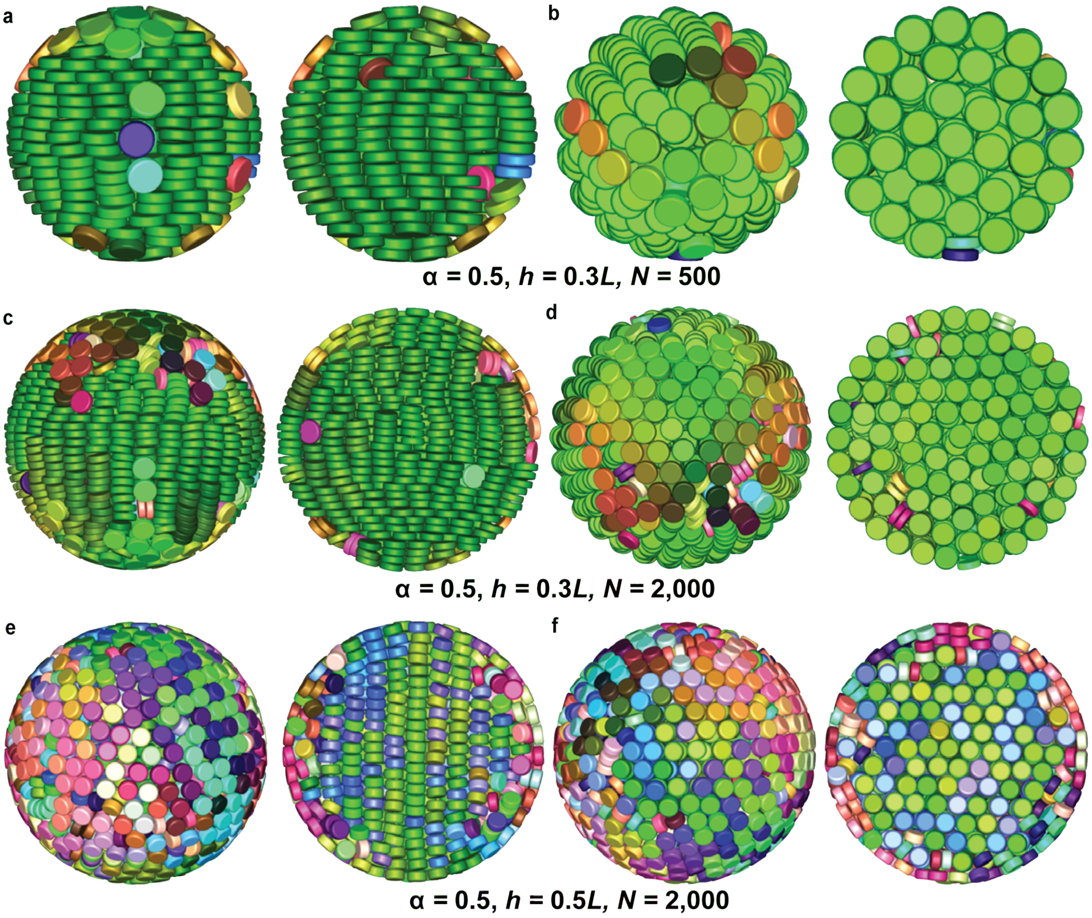

Supplementary Figure 9: Clusters obtained from computer simulations of hard disk-shaped particles with varying number of platelets. Fully compressed configurations as obtained from computer simulations with a,b) 500, c-d) 2,000 platelets with an aspect ratio of $h/L = 0.3$, and e-f) 2,000 platelets with an aspect ratio of $h/L = 0.5$ in spherical confinement. Different colours represent different particle orientations. The left and right column of each pair, shows a surface and cut-through view of each simulated cluster. For all platelets, $\alpha = 0.5$. For an interactive 3D view, see Supplementary Data 20, 21 and 22, respectively.

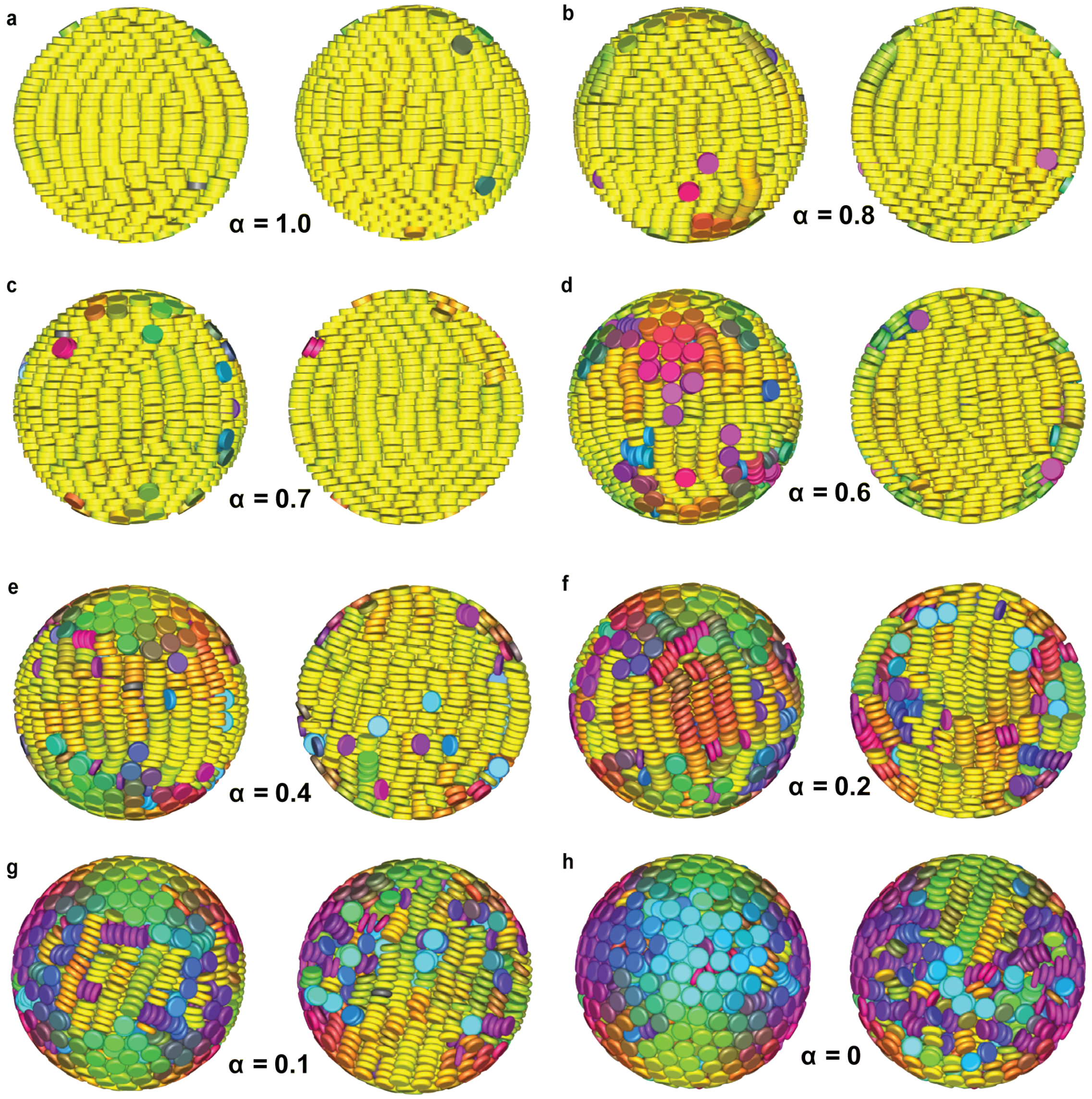

Supplementary Figure 10: Clusters from computer simulations composed of 2,000 platelets. Fully compressed configurations as obtained from computer simulations with 2,000 particles in spherical confinement with different shape parameters. a-h): $\alpha = 1.0$ (perfect flat cylinder), $0.8, 0.7, 0.6, 0.4, 0.2, 0.1$ and 0 (oblate hard spherocylinders, OHSCs), respectively. For all platelets, $h/L = 0.3$. Different colours represent different particle orientations. The left and right column of each pair, shows a surface (left column) and cut-through (right) view, respectively.

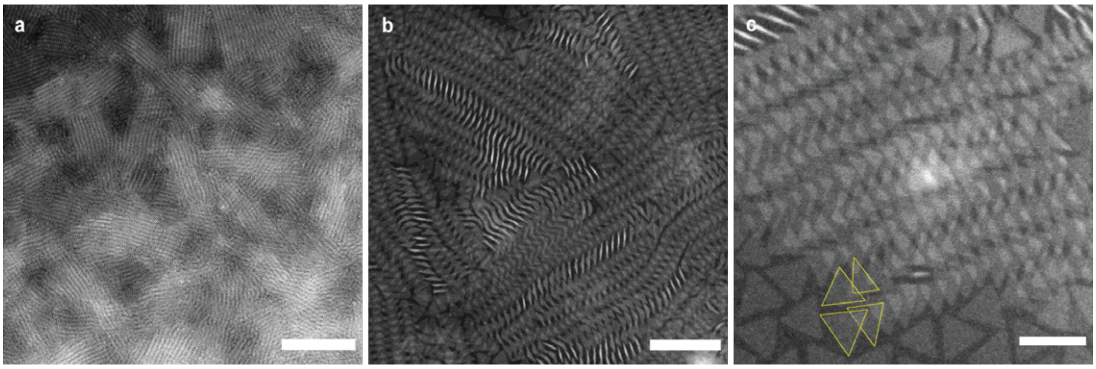

Supplementary Figure 11: 2D HAADF-STEM images of self-assembled superlattices composed of triangular LaF_3 NPLs. a) Multi-layered superlattice shows a ribbon-like structure. b,c) Interdigitation between columns can be visualised at different magnifications. The orientations of the NPLs are highlighted by the yellow triangles. Scale bars, a) 200 nm, b) 50 nm and c) 20 nm.

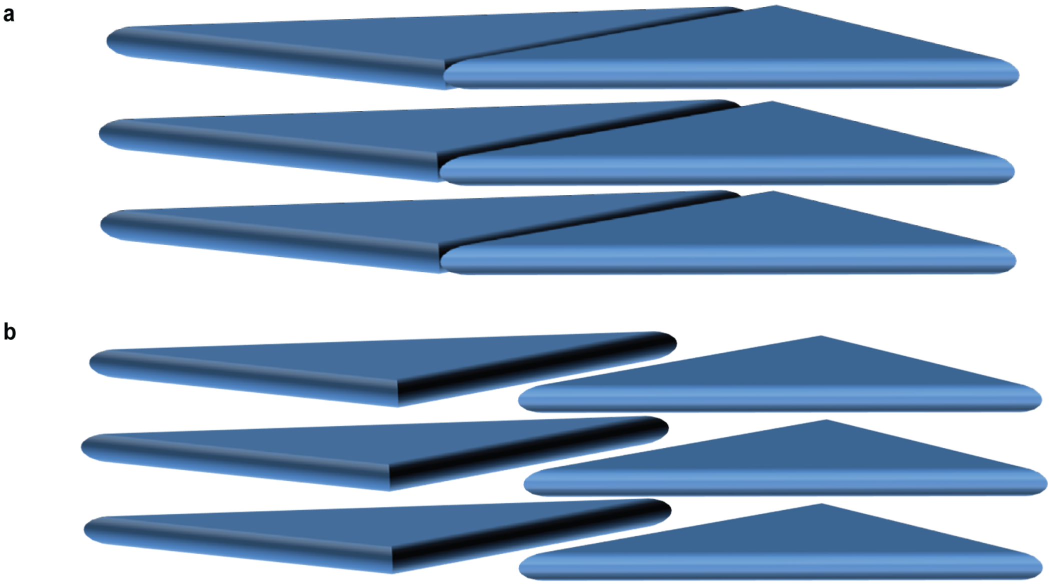

Supplementary Figure 12: Schematic illustration of two possible ways of stacking the equilateral triangular NPLs. Configuration of a) non-interlocked and b) interlocked columns.

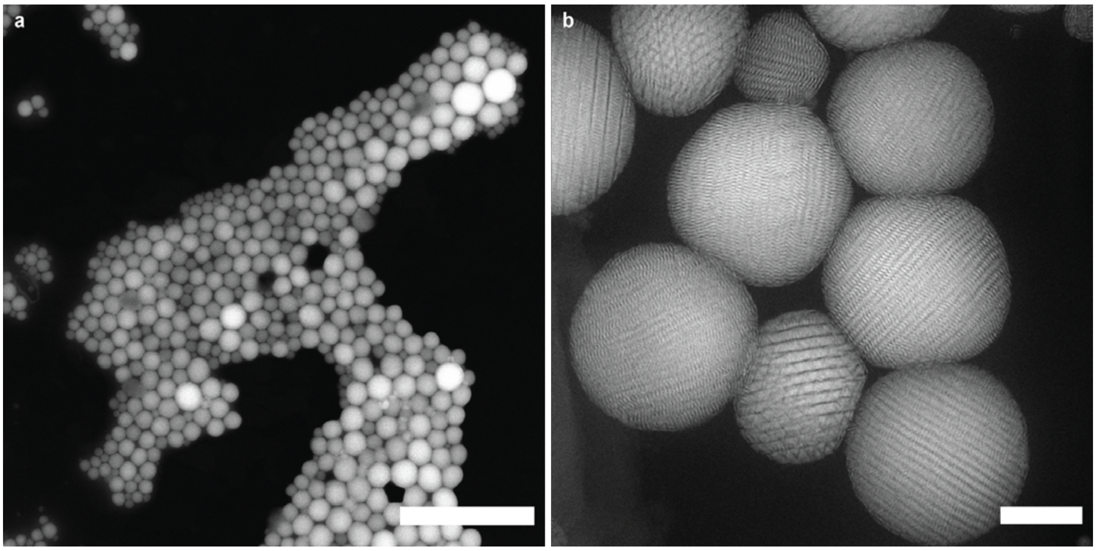

Supplementary Figure 13: Self-assembled SPs composed of triangular LaF_3 NPLs. a) Overview and b) a zoomed-in view of the self-assembled SPs. Scale bars, a) 2 μm and b) 100 nm.

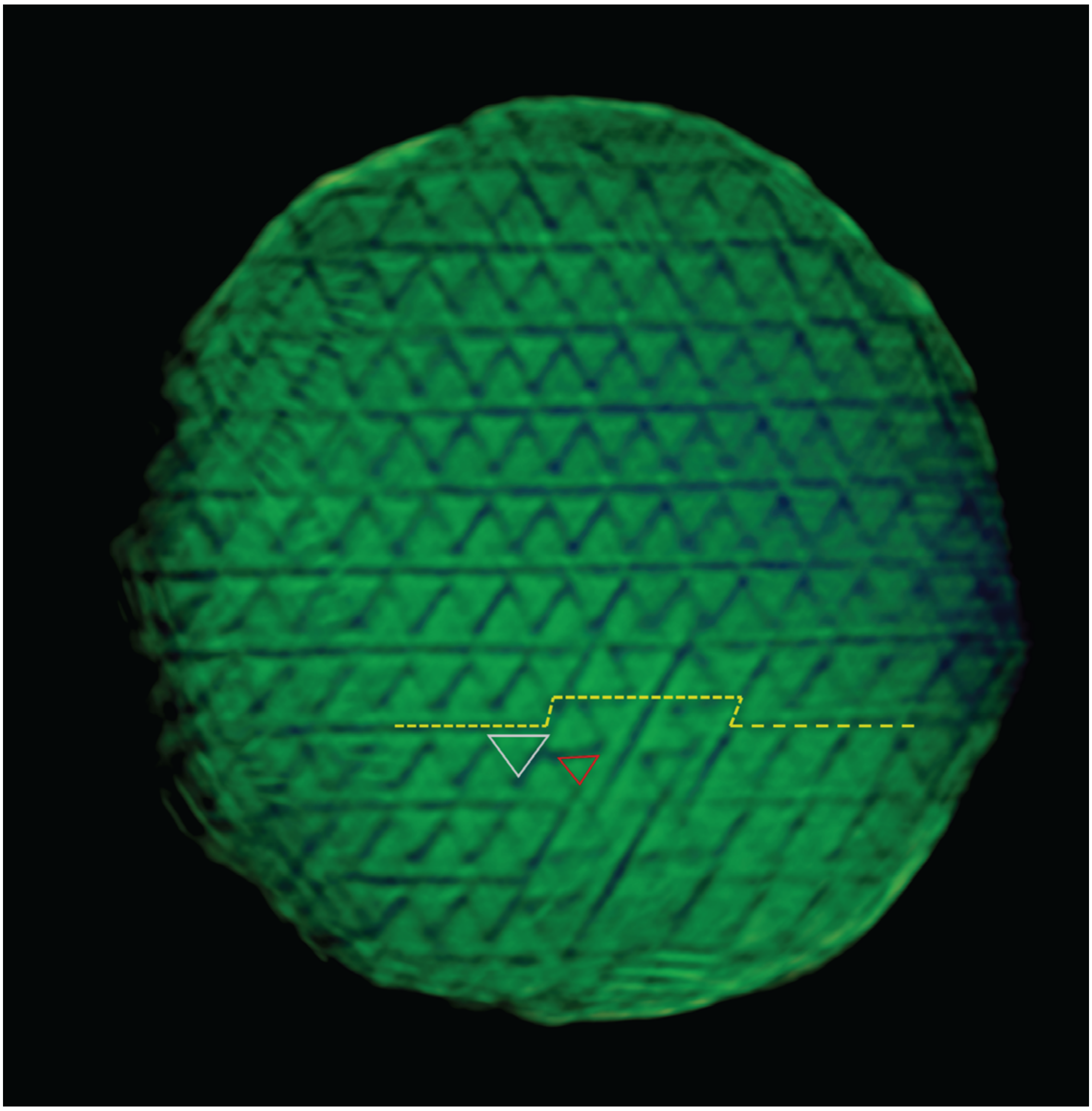

Supplementary Figure 14: Dislocations in a self-assembled LaF_3 SP. A dislocation (denoted as a yellow dashed line) is most probably induced by a slightly smaller-sized triangular LaF_3 NPL (denoted with a red triangle) compared to the rest (*e.g.*, denoted with a white triangle).

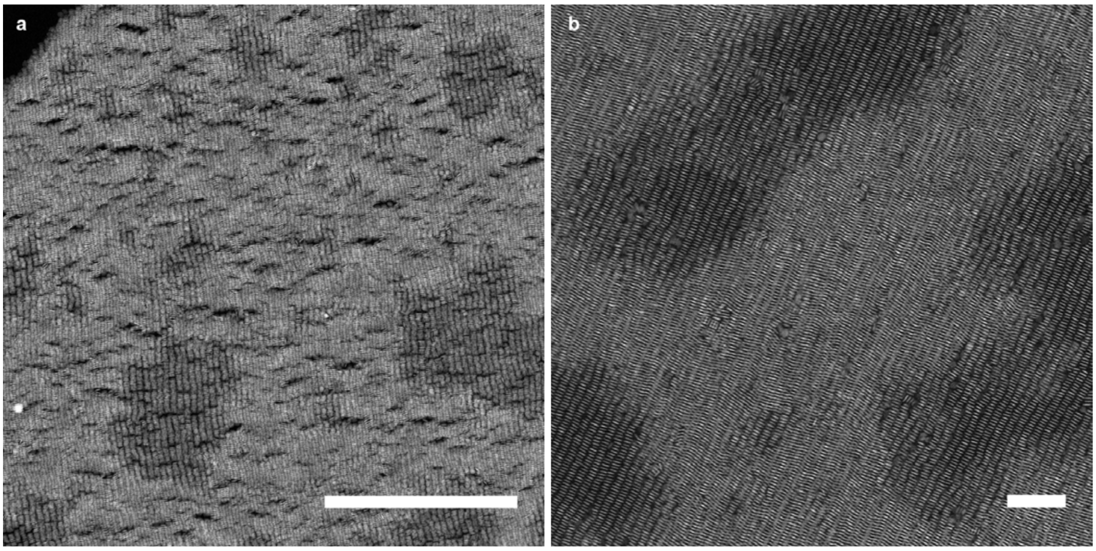

Supplementary Figure 15: 2D HAADF-STEM images of self-assembled superlattices composed of leaf-shaped GdF_3 NPLs viewed at different magnifications. a,b) Both single- and multilayered superlattices where the NPLs interdigitate with their neighbouring columns, showing a liquid crystalline structure. Scale bars, a) 500 nm and b) 100 nm.

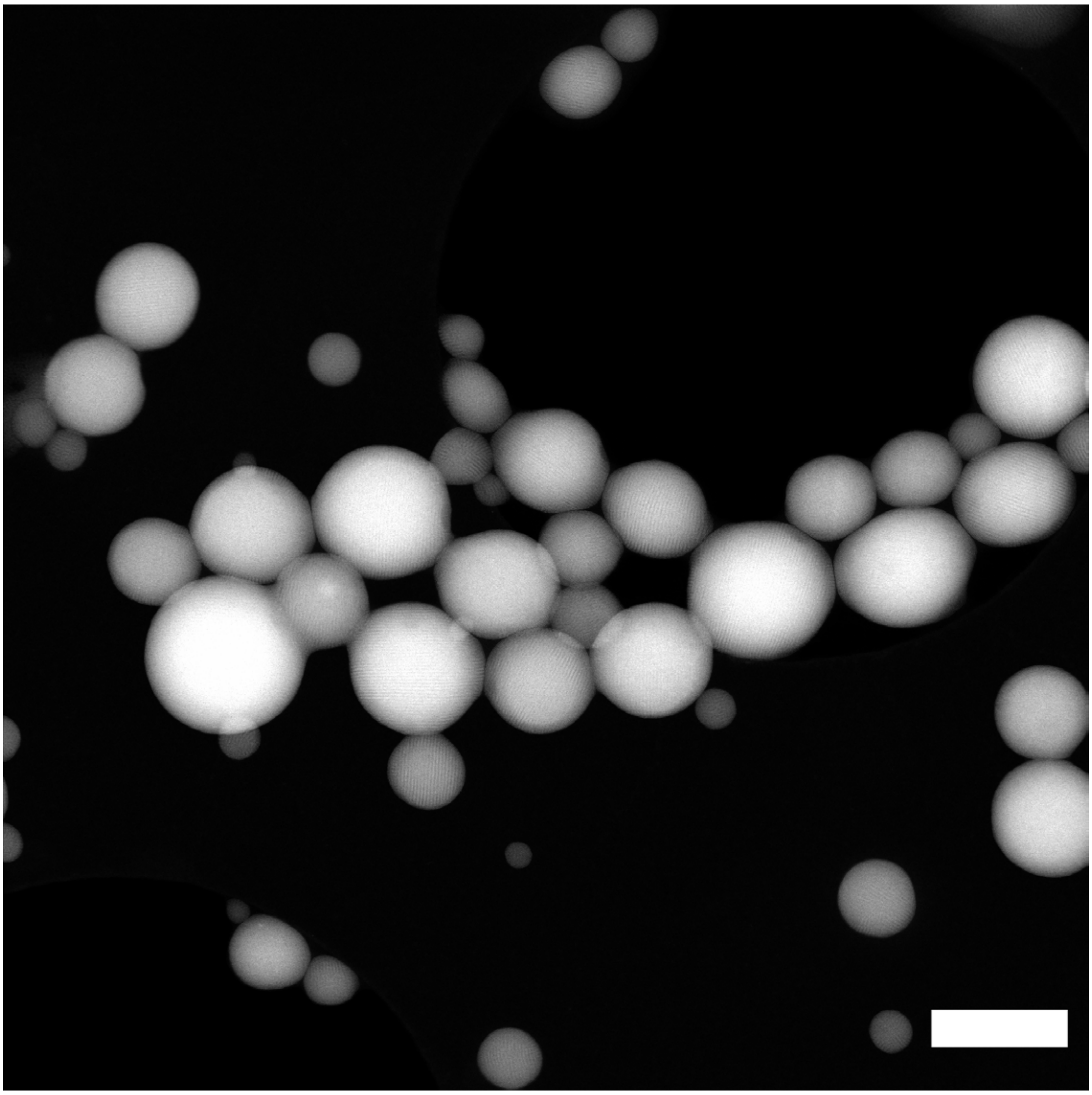

Supplementary Figure 16: Overview of self-assembled SPs composed of leaf-shaped GdF_3 NPLs. Scale bar, 500 nm.

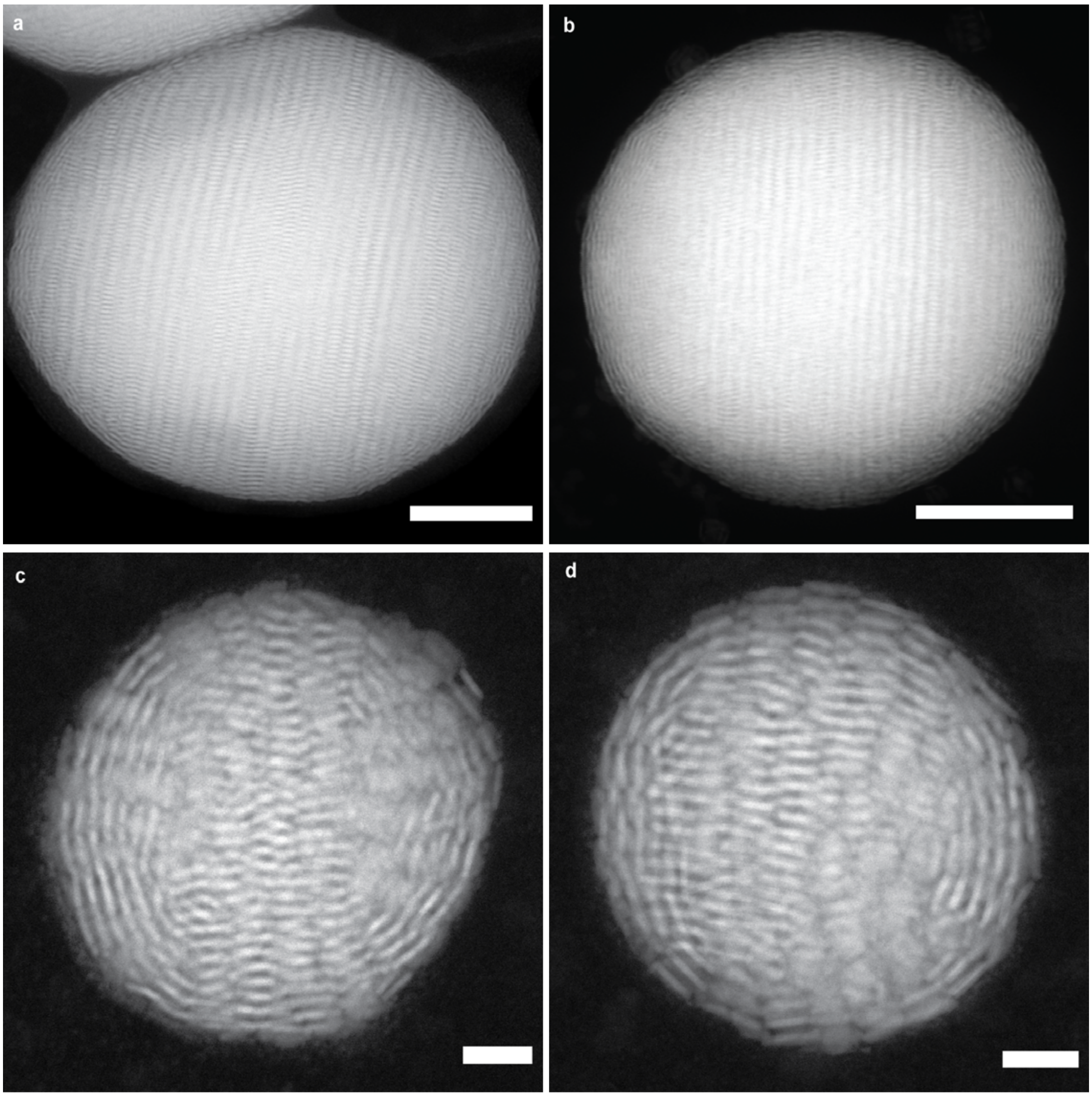

Supplementary Figure 17: SP size dependent column arrangement in the self-assembled SPs. 2D HAADF-STEM images of self-assembled SPs with a diameter of a) 428 nm, b) 307 nm, c) 127 nm and d) 119 nm, respectively. Scale bars, a,b) 100 nm, c,d) 20 nm.

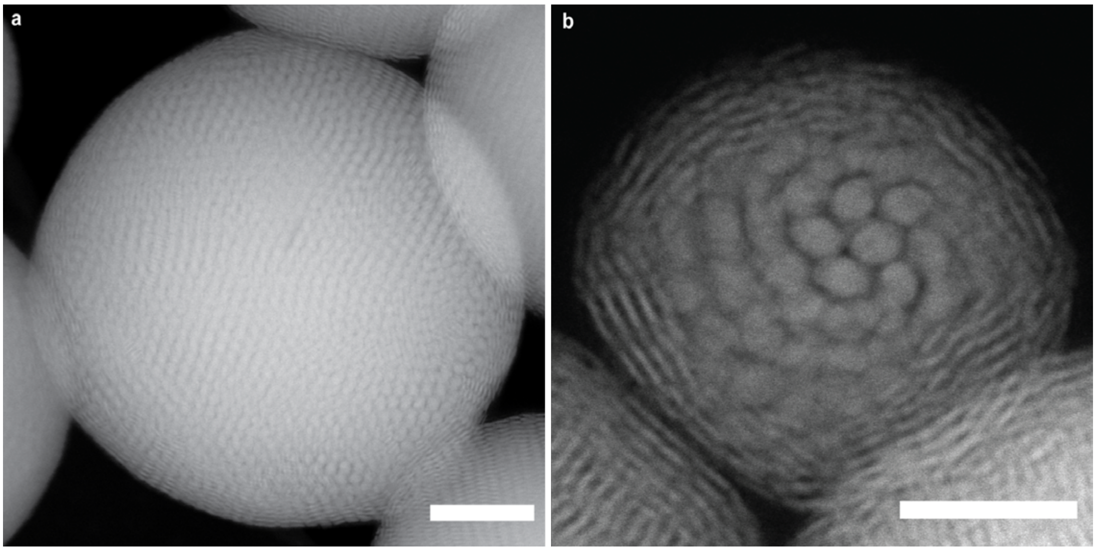

Supplementary Figure 18: Local hexagonal arrangement of the leaf-shaped GdF_3 NPLs. The leaf-shaped GdF_3 NPLs show a local hexagonal arrangement on the surface of a) a 470 nm and b) a 122 nm SP, respectively. Scale bars, a) 100 nm and b) 50 nm.

Supplementary Methods

Monte Carlo (MC) simulations of platelets were performed in the NVT ensemble. The particles were modeled as hard particles, the interaction potential $V = \infty$ when there is overlap and $V = 0$ otherwise. The overlap algorithm (https://github.com/Grieverheart/ntcd) that was used is a fast implementation of the Gilbert-Johnson-Keerthi (GJK) algorithm[1]. The rounded platelets were constructed by taking the Minkowski sum of a cylinder and a sphere. The particles were confined by a hard spherical shell of diameter R. The hard spherical shell was created from a spherical shell of overlapping cubes (we used roughly 1,900 cubes for the simulations with 1,000 particles to guarantee that the shell is smooth on the scale of the platelets). To simulate the compression the shell was decreased in size at a fixed rate. After each compression step all overlaps where removed (the number of MC moves needed to remove overlaps was always much smaller than the number of moves between compression steps). To aid the visualisation the platelets were coloured according to their orientation. The visualisation software that was used is Visual Colloids (https://github.com/michielhermes/viscol).

Supplementary Notes

Characteristic Eu^{3+} red emission which corresponds to the $^5D_0 \rightarrow {}^7F_J$ line emissions ($J = 0, 1, 2$) was observed in both samples, where the $^5D_0 \rightarrow {}^7F_1$ emission and $^5D_0 \rightarrow {}^7F_2$ emission are known as the magnetic dipole emission and electric dipole emission, respectively[2,3]. The relative intensity ratio between emission peaks in individual (free) disk-shaped EuF_3 NPLs dispersion and self-assembled SPs was changed slightly, such that the electric dipole emission was enhanced in the self-assembled SPs, and became roughly equal in intensity to the higher energy emissions. We speculate this was caused by changes in the local density of optical states in the dielectric spheres (*i.e.*, SPs in this case), which could for instance be further investigated by changing the refractive index of the suspension.

Supplementary References

1. Gilbert, E. G., Johnson, D. W. & Keerthi, S. S. A fast procedure for computing the distance between complex objects in three-dimensional space. *IEEE Robot. Autom.* **4,** 193–203 (1988).

2. Paik, T., Ko, D.-K., Gordon, T. R., Doan-Nguyen, V. & Murray, C. B. Studies of liquid crystalline self-assembly of GdF_3 nanoplates by in-plane, out-of-plane SAXS. *ACS Nano* **5,** 8322–8330 (2011).

3. Wang, Z., Senden, T. & Meijerink, A. Photonic effects for magnetic dipole transitions. *J. Phys. Chem. Lett.* **8,** 5689–5694 (2017).
