# Supplementary material for: Structural diversity in three-dimensional self-assembly of nanoplatelets by spherical confinement
Source: Nat Commun. 2022 Oct 12;13:6001. doi: 10.1038/s41467-022-33616-y (PMC9556815; doi:10.1038/s41467-022-33616-y)
Supplement: Supplementary file 3 — Description of Additional Supplementary Files [file 41467_2022_33616_MOESM3_ESM.pdf]

Description of Additional Supplementary Files

Supplementary Movie 1: Electron tomography reconstruction of a 160 nm-sized experimental supraparticle composed of 8,130 disk-shaped EuF_3 NPLs.

Supplementary Movie 2: Electron tomography reconstruction of a 183 nm-sized experimental supraparticle composed of disk-shaped EuF_3 NPLs.

Supplementary Movie 3: Electron tomography reconstruction of a supraparticle composed of triangular-shaped LaF_3 NPLs.

Supplementary Movie 4: Electron tomography reconstruction of a 180 nm-sized supraparticle composed of leaf-shaped GdF_3 NPLs.

Supplementary Movie 5: Electron tomography reconstruction of a 122 nm-sized supraparticle composed of leaf-shaped GdF_3 NPLs.

Supplementary Data 1: An experimental supraparticle composed of 8,130 EuF_3 disk-shaped nanoplatelets and its corresponding FFT pattern. The colour indicates the particle orientation.

Supplementary Data 2: A simulated supraparticle composed of 1,000 disk-shaped platelets with an aspect ratio of 0.5 and a roundness of 0.9. The colour indicates the platelet orientation.

Supplementary Data 3: A simulated supraparticle composed of 1,000 disk-shaped platelets with an aspect ratio of 0.5 and a roundness of 0.8. The colour indicates the platelet orientation.

Supplementary Data 4: A simulated supraparticle composed of 1,000 disk-shaped platelets with an aspect ratio of 0.5 and a roundness of 0.7. The colour indicates the platelet orientation.

Supplementary Data 5: A simulated supraparticle composed of 1,000 disk-shaped platelets with an aspect ratio of 0.5 and a roundness of 0.6. The colour indicates the platelet orientation.

Supplementary Data 6: A simulated supraparticle composed of 1,000 disk-shaped platelets with an aspect ratio of 0.5 and a roundness of 0.5. The colour indicates the platelet orientation.

Supplementary Data 7: A simulated supraparticle composed of 1,000 disk-shaped platelets with an aspect ratio of 0.5 and a roundness of 0.4. The colour indicates the platelet orientation.

Supplementary Data 8: A simulated supraparticle composed of 1,000 disk-shaped platelets with an aspect ratio of 0.5 and a roundness of 0.3. The colour indicates the platelet orientation.

Supplementary Data 9: A simulated supraparticle composed of 1,000 disk-shaped platelets with an aspect ratio of 0.5 and a roundness of 0.2. The colour indicates the platelet orientation.

Supplementary Data 10: A simulated supraparticle composed of 1,000 disk-shaped platelets with an aspect ratio of 0.5 and a roundness of 0.1. The colour indicates the platelet orientation.

Supplementary Data 11: A simulated supraparticle composed of 1,000 disk-shaped platelets with an aspect ratio of 0.5 and a roundness of 0 (perfect oblate hard spherocylinders). The colour indicates the platelet orientation.

Supplementary Data 12: A simulated supraparticle composed of 1,000 disk-shaped platelets with an aspect ratio of 0.1 and a roundness of 0.3. The colour indicates the platelet orientation.

Supplementary Data 13: A simulated supraparticle composed of 1,000 disk-shaped platelets with an aspect ratio of 0.2 and a roundness of 0.3. The colour indicates the platelet orientation.

Supplementary Data 14: A simulated supraparticle composed of 1,000 disk-shaped platelets with an aspect ratio of 0.3 and a roundness of 0.3. The colour indicates the platelet orientation.

Supplementary Data 15: A simulated supraparticle composed of 1,000 disk-shaped platelets with an aspect ratio of 0.4 and a roundness of 0.3. The colour indicates the platelet orientation.

Supplementary Data 16: A simulated supraparticle composed of 1,000 disk-shaped platelets with an aspect ratio of 0.6 and a roundness of 0.3. The colour indicates the platelet orientation.

Supplementary Data 17: A simulated supraparticle composed of 1,000 disk-shaped platelets with an aspect ratio of 0.7 and a roundness of 0.3. The colour indicates the platelet orientation.

Supplementary Data 18: A simulated supraparticle composed of 1,000 disk-shaped platelets with an aspect ratio of 0.8 and a roundness of 0.3 and corresponding FFT pattern, exhibiting an icosahedral symmetry. The colour indicates the platelet orientation.

Supplementary Data 19: A simulated supraparticle composed of 1,000 disk-shaped platelets with an aspect ratio of 0.9 and a roundness of 0.3 and corresponding FFT pattern, exhibiting an icosahedral symmetry. The colour indicates the platelet orientation.

Supplementary Data 20: A simulated supraparticle composed of 500 disk-shaped platelets with an aspect ratio of 0.3 and a roundness of 0.5. The colour indicates the platelet orientation.

Supplementary Data 21: A simulated supraparticle composed of 2,000 disk-shaped platelets with an aspect ratio of 0.3 and a roundness of 0.5. The colour indicates the platelet orientation.

Supplementary Data 22: A simulated supraparticle composed of 2,000 disk-shaped platelets with an aspect ratio of 0.5 and a roundness of 0.5. The colour indicates the platelet orientation.
