# Supplementary material for: Structural diversity in three-dimensional self-assembly of nanoplatelets by spherical confinement
Source: Nat Commun. 2022 Oct 12;13:6001. doi: 10.1038/s41467-022-33616-y (PMC9556815; doi:10.1038/s41467-022-33616-y)
Supplement: Supplementary file 4 — Supplementary Data 1 [file 41467_2022_33616_MOESM4_ESM.html]

Supplementary Data 1


## Supplementary Data 1

An experimental supraparticle composed of 8,130 EuF3 disk-shaped nanoplatelets and its corresponding FFT pattern. The colour indicates the particle orientation. The slider at the bottom can be used to visualise the inside. Click and drag to rotate.

Made using  Visual colloids.
