# Supplementary material for: Structural diversity in three-dimensional self-assembly of nanoplatelets by spherical confinement
Source: Nat Commun. 2022 Oct 12;13:6001. doi: 10.1038/s41467-022-33616-y (PMC9556815; doi:10.1038/s41467-022-33616-y)
Supplement: Supplementary file 9 — Supplementary Data 6 [file 41467_2022_33616_MOESM9_ESM.html]

Supplementary Data 6


## Supplementary Data 6

A simulated supraparticle composed of 1,000 disk-shaped platelets with an aspect ratio of 0.5 and a roundness of 0.5. The colour indicates the platelet orientation. The slider at the bottom can be used to visualise the inside. Click and drag to rotate.

Made using  Visual colloids.
