# Supplementary material for: Structural diversity in three-dimensional self-assembly of nanoplatelets by spherical confinement
Source: Nat Commun. 2022 Oct 12;13:6001. doi: 10.1038/s41467-022-33616-y (PMC9556815; doi:10.1038/s41467-022-33616-y)
Supplement: Supplementary file 22 — Supplementary Data 19 [file 41467_2022_33616_MOESM22_ESM.html]

Supplementary Data 19 


## Supplementary Data 19

A simulated supraparticle composed of 1,000 disk-shaped platelets with an aspect ratio of 0.9 and a roundness of 0.3 and corresponding FFT pattern, exhibiting an icosahedral symmetry. The colour indicates the platelet orientation. The slider at the bottom can be used to visualise the inside. Click and drag to rotate.

Made using  Visual colloids.
